# Gene profiling suggests a common evolution of bladder cancer subtypes

**DOI:** 10.1186/1755-8794-6-42

**Published:** 2013-10-17

**Authors:** Donna E Hansel, Zhongfa Zhang, David Petillo, Bin T Teh

**Affiliations:** 1 Department of Pathology, University of California at San Diego, 9500 Gilman Drive, MC 0612, La Jolla, CA 92093, USA; 2 Van Andel Research Institute, Grand Rapids, MI, USA; 3 National Cancer Centre of Singapore-VARI, Singapore, Singapore; 4 Duke-NUS Graduate Medical School, Singapore, Singapore

**Keywords:** Gene expression profiling, Bladder cancer, Urothelial, Squamous, Subtype

## Abstract

**Background:**

Bladder cancer exists as several distinct subtypes, including urothelial carcinoma (UCa), squamous cell carcinoma (SCCa), adenocarcinoma and small cell carcinoma. These entities, despite showing distinct morphology and clinical behavior, arise from the urothelial lining and are often accompanied by similar precursor/in situ findings. The relationship between these subtypes has not been explored in detail.

**Methods:**

We compared gene expression analysis of the two most common subtypes of bladder cancer, UCa (n = 10) and SCCa (n = 9), with an additional comparison to normal urothelium from non-cancer patients (n = 8) using Affymetrix GeneChip Human genome arrays (Affymetrix, Santa Clara, CA). The results were stratified by supervised and unsupervised clustering analysis, as well as by overall fold change in gene expression.

**Results:**

When compared to normal urothelium, UCa showed differential expression of 155 genes using a 5-fold cut-off. Examples of differentially regulated genes included topoisomerases, cancer-related transcription factors and cell cycle mediators. A second comparison of normal urothelium to SCCa showed differential expression of 503 genes, many of which were related to squamous-specific morphology (desmosomal complex, intermediate filaments present within squamous epithelium, squamous cornifying proteins, and molecules upregulated in squamous carcinomas from other anatomic sites). When compared, 137 genes were commonly dysregulated in both UCa and SCCa as compared to normal urothelium. All dysregulated genes in UCa were shared in common with SCCa, with the exception of only 18 genes. Supervised clustering analysis yielded correct classification of lesions in 26/27 (96%) of cases and unsupervised clustering analysis yielded correct classification in 25/27 (92.6%) of cases.

**Conclusions:**

The results from this analysis suggest that bladder SCCa shares a significant number of gene expression changes with conventional UCa, but also demonstrates an additional set of alterations that is unique to this entity that defines the squamous phenotype. The similarity in deregulated gene products suggests that SCCa may be a much more closely related entity at the molecular level to conventional UCa than previously hypothesized.

## Background

**Figure 1:**
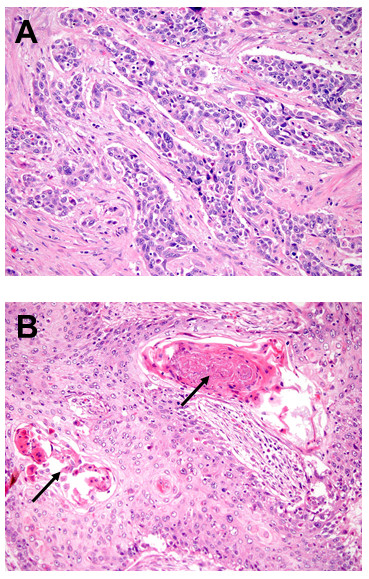
Two major subtypes of bladder cancer include (A) urothelial carcinoma, which is the most common form of bladder cancer and contains variably sized nests and (B) squamous cell carcinoma, which is characterized by desmosomes and keratin pearls (arrow).

Urothelial carcinoma (UCa) represents the most common form of bladder cancer in the United States (>90%) and is characterized by frequent mutations in *TP53*, *RB* and *PTEN*[1]. Morphologically, UCa consists of invasive nests of carcinoma cells with variable atypia and frequent surrounding retraction artifact (Figure 1A), although this appearance can vary significantly. Less common forms of bladder cancer in the United States include squamous cell carcinoma (SCCa; Figure 1B), adenocarcinoma and small cell carcinoma, which are defined as pure morphologic entities that lack a typical urothelial component [2]. Our understanding of the molecular relationships between these other forms of bladder cancer that arise from the urothelial lining has been limited and may be based on the low number of cases available for study and/or lack of significant attention paid to this topic. It appears, however, that despite a similar origin from the surface urothelium, these various forms of bladder cancer show differential clinical behavior, morphologic appearances, immunohistochemical markers and response to chemotherapy.

SCCa represents the second most common form of bladder cancer in the United States (5-8% of bladder cancer cases) and is distinguished by invasive squamous carcinoma cells containing desmosomes and often keratin formation [3]. A higher incidence of bladder SCCa has been reported in parts of the Middle East and Africa, however, where chronic infection with the water-bourne parasite *Schistosoma haematobium*, represents a major risk factor in the development of this disease [4]. Other potential risk factors in the development of SCCa include long-term catheterization, calculi that form in the urinary tract, and a nonfunctioning bladder, amongst others, and these risk factors may account for many instances of SCCa identified in the United States and Europe.

Despite the distinct appearance of UCa and SCCa, the cellular origin of these two forms of bladder cancer has been debated. Whereas UCa can arise in association with surface high-grade changes of the urothelium (e.g., carcinoma in situ) and SCCa can arise in association with squamous dysplasia and squamous carcinoma in situ of the bladder, there are numerous cases that show overlap of surface changes. For example, SCCa can occur in the absence of any surface squamous metaplasia and may only be associated with urothelial carcinoma in situ [3]. Furthermore, UCa itself has been shown to demonstrate “divergent” differentiation with the ability of UCa tumor cells to develop squamous or glandular features. These findings suggest that 1) the molecular relationship between historically distinct subtypes of bladder cancer may be more similar than previously hypothesized and 2) the surface urothelium in a bladder undergoing neoplastic alterations may be unusually suited to give rise to divergent phenotypes in the setting of both in situ and invasive disease.

To date, much of the molecular analysis on bladder SCCa has been limited due to a predominant focus on infectious, Schistosomal-derived cases as well as use of immortalized bladder cancer cell lines in a number of studies [5-8]. In this setting, the understanding of the relationship between UCa and SCCa, as well as the distinction between primary molecular changes and those secondarily induced by infection-specific responses, becomes difficult. We sought to compare the two most common forms of pure bladder cancer in the US (UCa and non-infectious SCCa) using multi-level gene expression analysis to determine the relationship and possible hierarchy of these two examples of bladder cancer. The results from our study suggest a closer relationship between these neoplastic entities than previously proposed; a shared evolution of these cancers may represent an opportunity for targeting bladder cancer along common pathways early in the disease process.

## Methods

### Specimen collection

Specimens were collected with Institutional Review Board approval. The existing bladder cancer biobank (1971 onwards) was searched for snap-frozen tissue obtained from non-neoplastic bladder and/or ureter and from patients with either UCa or SCCa. Frozen sections were obtained from all specimens and reviewed; specimens with any necrosis or <90% tumor or normal cell nuclei were excluded from analysis. H&E slides corresponding to the initial pathology specimen associated with each sample were re-reviewed for accuracy of tumor classification. The clinical records for any patients with normal urothelium were reviewed; any patient with a precedent or subsequent occurrence of urinary tract neoplasia was excluded from analysis. This resulted in 8 normal urothelium specimens, 10 UCa specimens and 9 SCCa specimens (total in archive) used for analysis. No patient with SCCa had a precedent or concurrent history of Schistosomal infection. This study was approved by the Cleveland Clinic IRB.

### Raw gene expression levels

Ten micrograms of total RNA from each sample was processed using the Affymetrix GeneChip one-cycle target labeling kit (Affymetrix, Santa Clara, CA). The resultant biotinylated cDNA was fragmented and subsequently hybridized to the GeneChip Human genome (54,675 probe sets including more than 35,000 human genes; Affymetrix). Arrays were washed, stained, and scanned using the Affymetrix Model450 Fluidics Station and Affymetrix Model 3000 scanner per manufacturer’s recommended protocols.

Expression values were generated using Microarray Suite (MAS) v5.0 software (Affymetrix). The probes were redefined according to a new study (http://brainarray.mhri.med.umich.edu/Brainarray/Database/CustomCDF/version10) to combine probes representing the same gene for a single profile per gene. The hybridizations were normalized using the robust multichip averaging (rma) algorithm in the Bioconductor package *affy* (see http://www.bioconductor.org/) in order to obtain summary expression values for each probe set [9,10]. This resulted in more than 17,000 genes, each of which then has one numeric number to represent its relative gene expression intensity in the sample.

### Clustering study

A hierarchical clustering algorithm was used to identify unsupervised clusters based on the Euclidean distance for dissimilarities between the data samples. The slightly modified “plot.phylo” program from analyses of phylogenetics and evolution (ape) package of R was used to show the clustering results [11,12]. The interquartile range (IQR) and coefficient of variation (CV) were used to filter identified genes in the unsupervised clustering study. IQR was defined to be the distance between the third and first quartiles of the data; the CV of a vector was defined to be the standard deviation divided by its mean value. We used IQR > 0.3 and CV > 0.05 as our filtering criteria. This resulted in a data set of approximately 13600 genes. Other cutoff values provided similar clustering results. We also used the limma package to identify genes for supervised clustering analysis. When more than two classes of genes were present in the study group, the comparison was made between *all pairs* of classes. When comparison was made between two conditions, we used a fold change of 5 as a cutoff value to declare a gene significant.

We set 0.05 as our significance level for all tests. All calculations were implemented in R environment (R > 2.15.0, see http://www.r-project.org).

## Results

### Comparative analysis

**Figure 2:**
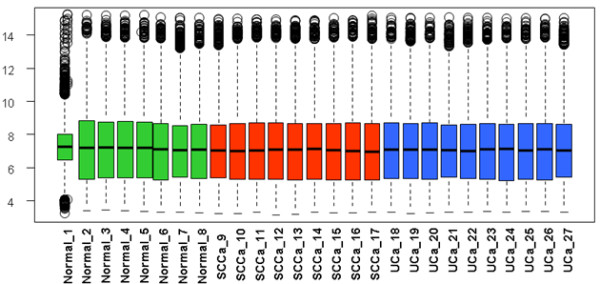
Boxplot of the 27 samples used for study presented in log2 scale.

Despite the shared urothelium from which SCCa and UCa arises, it is unclear whether these two morphologically distinct forms of bladder cancer share significant molecular overlap and, if so, whether a hierarchy in tumor types exists. In order to address this question, we performed a four-way interrogation of gene expression profiles: 1) normal urothelium versus SCCa, 2) normal urothelium versus UCa, 3) normal urothelium versus SCCa and UCa combined (shared alterations) and 4) UCa versus SCCa (divergent alterations). We included for analysis 8 samples of normal urothelium, 10 samples of invasive high-grade UCa and 9 samples of invasive SCCa. A boxplot of the data set shows that all samples have a roughly comparable distribution of the gene expression values, except only one sample (normal sample 1; Figure 2). When analyzed by subsequent unsupervised or supervised clustering studies, sample 1 did correctly segregate into the normal urothelial cluster; we therefore retained this sample in our study set.

**Figure 3:**
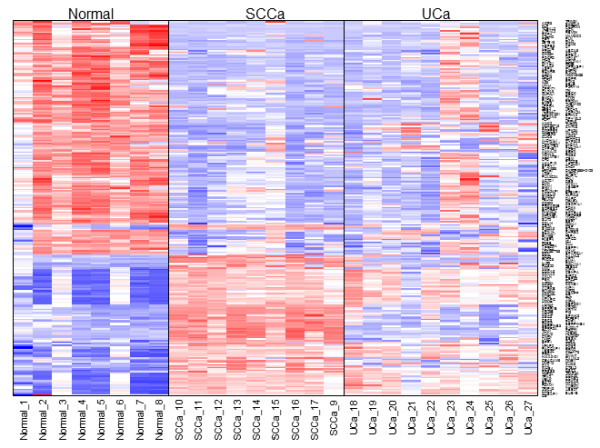
Heatmap comparing UCa and SCCa to normal urothelium for all 27 samples using supervised clustering and a fold change of 5, with 262 genes represented.

**Figure 4:**
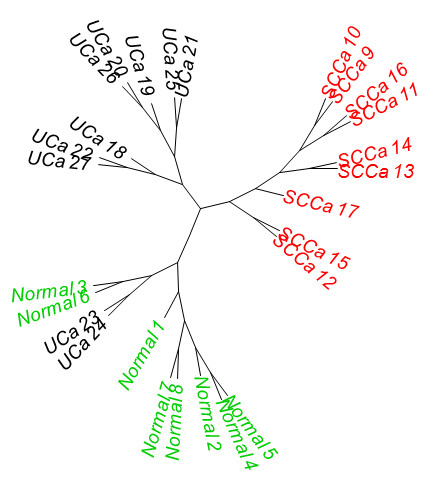
Supervised clustering of the 27 samples using the top most significant 877 genes (limma method from Bioconductor using adjusted p value <=0.001; error rate 1/27=3.7%; black = UCa; red = SCCa; green = normal).

**Figure 5:**
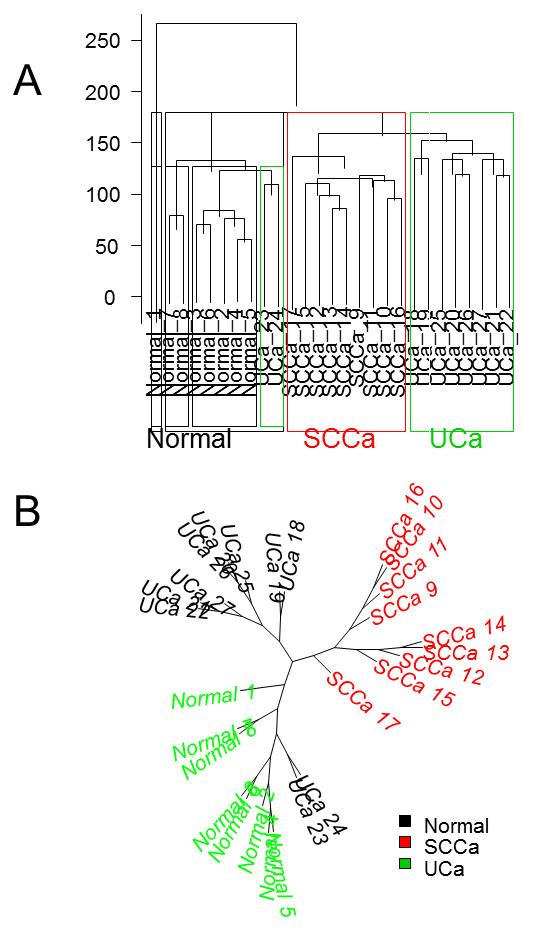
Unsupervised clustering analysis using approximately 4500 genes filtered first by the interquantile range (IQR, >0.3) then by the coefficient of variation (CV, >0.05) resulted in an error rate of 2/27=7.4% (black = UCa; red = SCCa; green = normal) represented as (A) dendrogram and (B) phylogram (unrooted).

Unexpectedly, the gene expression profiles revealed a large number of shared gene expression differences in UCa and SCCa relative to the normal urothelium when using a 5-fold cut-off (n = 137, Figure 3). In addition to these shared gene expression differences, SCCa demonstrated an additional 366 uniquely dysregulated genes relative to normal urothelium, whereas UCa demonstrated only an additional 18 genes that were uniquely dysregulated relative to normal urothelium. Using supervised clustering (Figure 4) and unsupervised clustering (Figure 5A, B) analysis, we were able to reproducibly segregate normal urothelium, UCa and SCCa specimens, although two specimens (UCa23 and UCa24) appeared slightly different than other tumors in the UCa category, but could correctly segregate with other UCa specimens when a lower threshold value was applied to the analysis; specifically, no morphological difference was appreciated in these two specimens.

**Figure 6:**
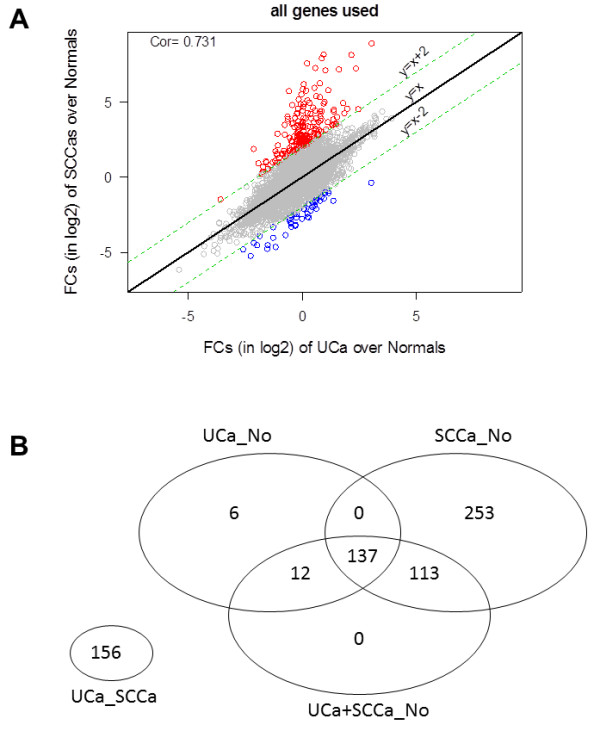
Significantly differentially expressed genes between UCa and SCCa versus normal samples represented as (A) scatterplot comparing fold change (FC) differences between UCa versus normal samples and SCCa versus normal samples (98.7% of genes have FC ≤2, correlation coefficient=0.73) and (B) Venn diagram with FC cutoff value=5.

All differentially expressed genes were used to obtain fold changes (in log2 scale) to compare UCa versus normal and SCCa versus normal (Figure 6A). The majority of genes have fold change differences within 2 (17,468, 99.25%, grey). A relatively larger number of genes have fold change differences above 2 (184, red) than the number of genes with fold change differences below -2 (47, blue). Overall, the fold change vectors correlated well with each other (cor. coefficient greater than 0.73), with the exception of the 184 genes located above the selected area (red), which are significantly higher in SCCa when compared to normal urothelium. A summary of the 4-way analysis performed with total gene expression differences is presented in Figure 6B.

### Commonly dysregulated genes in UCa and SCCa versus normal urothelium

We next sought to determine commonalities in gene expression changes in UCa and SCCa versus normal urothelium. As normal urothelium lines the urinary tract throughout its length, and represents the common epithelium from which any form of bladder cancer (with the exception of urachal carcinoma) derives, we queried whether shared pathways were commonly altered in these forms of bladder cancer. Using this rationale, we identified 137 genes that differed by at least 5-fold in cancer specimens relative to normal urothelium (45 upregulated and 92 downregulated genes), with a representative subset containing functions related to cell growth and/or reported in cancer listed in Table 1.

**Table 1:** Commonly dysregulated genes shared by urothelial and squamous carcinoma versus normal rothelium

**Gene**	**Fold change UCa**	**Fold change SCCa**	**Function**
UBE2C	15.1	15.1	Ubiquitin-conjugating enzyme; promotes cell cycle; amplified in UCC
TOP2A	13	14.8	DNA topoisomerase
CDC20	11.4	21.1	Regulates RUNX1; interacts with Aurora A; cell cycle regulation
CCNB2	9.49	12.5	Cyclin B2; TGF-beta mediated cell cyle control
CEP55	9.32	15.1	Centromeric protein regulated by FOXM1
TPX2	9.32	11.6	Microtubule formation at kinetochores
TTK	9.08	10.7	Mitotic spindle checkpoint protein; smoking responsive gene
FOXM1	8.99	7.66	Can increase levels of cyclin family members; smoking responsive gene
ZWINT	8.87	7.37	Kinetochore interactor
BUB1B	8.83	9.06	Kinase involved in spindle checkpoint function
MELK	8.58	12.4	AMPK related kinase; implicated in stem cell function
DLG7	7.98	12.3	Putative stem cell factor that interacts with kinetochore; increased in UCC
NUF2	7.64	7.97	Kinetochore-associated protein involved in chromosome segregation
MAD2L1	7.61	9.06	Spindle checkpoint protein; regulates start of anaphase
AURKB	6.62	9.53	Microtubule-associated protein involved in chromosome segregation
CCNB1	6.43	9.89	Cyclin B1; complexes with p34 to regulate mitotic activity
RACGAP1	6.24	5.89	RhoGTPase encoding gene
AURKA	5.64	8.51	Kinetochore-associated protein found at spindle poles during mitosis
ADH1B	-42.3	-70.1	Involved in tobacco smoke detoxification; implicated in esophageal cancer
UPK1A	-14.4	-22.4	Urothelium-associated protein
FHL1	-12.5	-8.37	LIM-protein that regulates apoptosis and proliferation
ANXA10	-12.1	-18.8	Regulates cell growth; synergizes with p53 mutation for worse outcomes
ADAMTS1	-10.0	-8.56	Matrix metalloproteinase
DARC	-9.93	-15.9	Binds cytokines; can influence tumor cell binding to endothelial cells
MYH11	-8.96	-18.8	Smooth muscle myosin
DMN	-8.81	-11.0	Intermediate filament associated with desmin
TCF21	-7.43	-9.92	Tumor suppressor gene; undergoes methylation in some cancers
HLA-DQA1	-7.01	-8.38	Expression may be altered following repeated BCG exposure
CD69	-6.63	-5.48	Lectin superfamily; reduced in head and neck SCC patients
ANK2	-6.28	-8.84	Links integral membrane proteins to the underlying cytoskeleton
CLU	-6.18	-5.72	Apoptotic mediators; expression decreased in many cancers
SELE	-6.14	-7.75	Inflammatory mediator; regulates immune cell-endothelial interaction
JAM2	-6.07	-7.61	Tight junction protein; regulates immune cell binding
AOX1	-5.76	-7.12	Nicotine metabolizing protein
PROM1	-5.27	-8.29	Expressed in adult stem cells; mediates differentiation
CCL14	-5.09	-8.40	Cytokine

Differentially expressed genes have an adjusted p value of <0.05.

The mitotic spindle checkpoint appeared generally upregulated, with overexpression of gene products of aurora kinase A (*AURKA*), aurora kinase B (*AURKB*), *BUB1B*, *NUF2*, *MAD2L1*, *CCNB1*, *TPX2*, *ZWINT*, *ZWINT* and *CDC20*. Although these genes may be upregulated simply due to increased proliferative capacity of carcinomas, aurora kinase A has been previously investigated in UCa, where it is commonly found to be amplified [13] and may be a potential novel therapeutic target [14], which validates our results. A second category of upregulated genes included nicotine-responsive genes, as identified in both lung and head and neck squamous cancers, and include *TTK*, *CEP55*, *PRC1* and *FOXM1*[15-18]. As tobacco smoking is a preeminent risk factor for the development of both UCa and SCCa of the bladder, these genes may reflect this association. An additional category of overrepresented gene products included putative stem cell markers and/or mediators encoded by *TTK*, *MELK*, *DLG7*, and *PBK*[19-22]. Of note, very few pro-migratory factors were found to be upregulated in this shared population with the most likely pro-migration factor represented by *RACGAP1*, which encodes a small RhoGTPase.

Downregulated genes grouped into the major categories of inflammatory mediators [23], nicotine metabolizing genes, regulators or apoptosis and cell adhesion factors. Downregulated gene products include *CFD*, *C7*, *DARC*, *PTX3*, *CD302*, *HLA-DQA1*, *CD69*, *P2RY14*, *SELE*, *JAM2*, and *CCL14*, which include mediators of inflammatory cell adhesion, humoral response and monocyte activity. *HLA-DQ1* has been evaluated previously in UCa and its expression is associated with repeated exposure (and response) to BCG [24].

*ADH1B* and *AOX1* are involved in the metabolism of nicotine, with the former gene implicated in the risk of esophageal carcinoma [25]. Apoptotic mediators that are downregulated include *CLU*, *FHL1* and *PCP4*, whereas cell adhesion and cytoskeletal mediators that are downregulated include *UPK1A*, *MYH11*, *DMN*, *MFAP4*, *ITM2A*, *ANK2*, *JAM2*, *MYLK*, *PROM1*, *DPT*, and *FBLN5*.

Of the 137 genes differentially expressed between bladder UCa and SCCa versus normal urothelium, 18 have been previously reported to be up/down regulated in UCa and 35 have been reported in SCCa arising from non-bladder sites. Due to the rarity of profiling papers available on bladder SCCa, however, these factors have not been studied in this entity to date.

### A limited subset of uniquely dysregulated genes defines UCa

One of the most surprising results from this study are the very small number of genes that were found to be uniquely dysregulated in UCa versus normal urothelium (n = 18; representative subset Table 2). The remainder of dysregulated genes (n = 137) are found in common with those altered in bladder SCCa. Uniquely dysregulated genes in UCa include *CLCA4* (chloride channel), *IL33*, *GPR171* (G protein coupled receptor), *CENPF* (centromere protein F) and *CD36* (thrombospondin receptor). *EZH2* has been reported to be upregulated in UCa and represents a putative stem cell marker [26] and a repressor of E-cadherin expression [27]; of relevance, E-cadherin is frequently lost in high-grade UCa [28].

**Table 2:** Uniquely dysregulated genes in grothelial garcinoma versus normal urothelium

**Gene**	**Fold change UCa**	**Function**
MEST	8.14	Encodes a member of alpha/beta hydrolase superfamily; imprinting in cancer
EZH2	5.57	Stem cell related gene; previously reported as upregulated in UCa
CENPF	5.54	Component of centromere/kinetochore complex; affects chromosome segregation
GINS2	5.43	Psf2 homolog; complex component that regulates DNA replication in yeast
CLCA4	-11.9	Member of the calcium sensitive chloride conductance superfamily
POU2AF1	-8.96	Regulates TH1 and TH2 immune responses
IL33	-7.99	Member of IL1 family; enhances TH2 cytokine production
SEPP1	-6.94	Selenoprotein; may affect oxidative stress response
IL6	-6.57	Inflammatory cytokine; increased after BCG administration in the bladder
GPR171	-6.45	G protein-coupled receptor; may regulate hematopoietic progenitor cells
FOSB	-6.26	One member of the AP-1 transcription factor complex; implicated in ovarian carcinoma
ITK	-6.05	Regulator of T cell proliferation
AGR3	-5.62	May regulate protein folding; may regulate cisplatin resistance in ovarian cancer
CCL19	-5.45	Cytokine affecting B and T cell migration; may enhance B-cell mediated immunity;
CD36	-5.42	Thrombospondin receptor; may affect tumor vascularity and matrix content
DKK1	-5.33	Inhibits WNT signaling; may inhibit invasive behavior and self-renewal in some cancers

Differentially expressed genes have an adjusted p value of <0.05.

### Well-categorized squamous factors are uniquely upregulated in SCCa

Finally, we analyzed uniquely dysregulated genes in SCCa versus normal urothelium and identified 185 upregulated and 181 downregulated unique genes that differed by at least 5-fold between these two groups (representative subset presented in Table 3). The majority of dysregulated genes are factors that have been associated with the squamous phenotype and histology, with many of these factors identified in squamous carcinomas arising at other sites. Upregulated gene products include keratins that are specific for squamous epithelium (*KRT6B*, *KRT16*, *KRT5*, *KRT20*), the family of S100 calcium binding proteins commonly upregulated in SCCa from various anatomical sites (*S100A7*, *S100A8*, *S100A9*, *S100A12*, *S100A14*, *S100A16*), the serpin family (*SERPINB1*-*7*), desmosome-associated proteins that characterize squamous epithelium (*DSG1*, *DSG3*, *PKP1*, *PKP3*), numerous peptides (*PI3*, *SPINK5*, *KLK7*, *SLPI*), and a variety of pro-motility factors. Downregulated gene products include putative tumor suppressor genes (*SCUBE2)*, factors previously reported as lost in aggressive bladder cancer (*FOXA1*, *GATA3*, *UPK3A*), and metabolizing enzymes with polymorphisms affecting cancer risk (*UGT1A10*, *UGT1A7*).

**Table 3:** Uniquely dysregulated genes in squamous cell carcinoma versus normal urothelium

**Gene**	**Fold change SCCa**	**Function**
KRT6B	289	High molecular weight cytokeratin; stratified epithelium differentiation
S100A7	242	Promotes migration and invasion of SCCa; reported in urine of bladder SCCa patients
KRT16	147	High molecular weight cytokeratin
PI3	140	Peptidase inhibitor 3/elafin; marker of abnormal squamous growth; induced by inflammation
DSG3	77.6	Desmosome-associated protein; upregulated in various forms of SCCa
SERPINB4	58.3	Member of human SCCa antigen locus; activated by STAT3; enhances survival of SCCa cells
CNFN	56.3	Cornifelin; squamous epithelial marker
KLK10	53.9	Serine protease; increased in oral and lung SCCa
SERPINB3	42.5	Member of human SCCa antigen locus; activated by STAT3; enhances survival of SCCa cells
FGFBP1	31.3	FGF carrier protein; promotes angiogenesis in SCCa from various sites
MMP1	31.0	Matrix metalloproteinase that degrades collagens; induced by EGF in bladder cancer patients
DSG1	29.6	Desmosome-associated protein; enhances loss of cell adhesion in SCCa from various sites
KRT5	25.8	High-molecular weight cytokeratin; expressed by squamous epithelium
DSC3	25.1	Desmosome-associated protein; marker of squamous differentiation; can inhibit EGFR pathway
S100A8	22.9	May predict metastatic potential of bladder cancer; increased in SCCa from various sites
LY6D	21.1	Affects interaction between SCCa cells and endothelial cells
CA2	19.3	Carbonic anhydrase II; Previously reported in bladder SCCa
MMP10	18.7	Matrix metalloproteinase; activated by EGF and STAT3; inhances SCCa invasion at other sites
PTHLH	15.5	Parathyroid hormone-like hormone; previously reported in bladder SCCa; may affect apoptosis
SCEL	15.1	Squamous epithelium marker
CALB1	12.9	Calbindin 1; calcium binding protein of the troponin C superfamily
PTPRZ1	11.9	Receptor protein tyrosine phosphatase; involved in CNS development
LAMC2	8.95	Laminin, gamma2; Overexpressed in esophageal SCC
KIF14	6.58	Microtubule motor protein; reported in laryngeal carcinoma
JUP	6.31	Associated with both desmosomes and intermediate junctions
SCUBE2	-27.1	Secreted protein; putative tumor suppressor in breast cancer
HPGD	-20.6	Metabolism of prostaglandins; downregulated in gastric cancer by COX2
HMGCS2	-15.6	HMG-CoA synthase family; enzyme involved in ketogenesis
CYP4B1	-15.0	Involved in drug metabolism and lipid synthesis; related to bladder cancer risk in one study
TGFBR3	-13.1	Encodes TGF-β receptor III; reduced expression in numerous cancers
UPK3A	-10.9	Uroplakin 3A; urothelial marker; loss of expression associated with aggressive bladder cancer
AOC3	-10.1	Copper amine oxidase; aids in leukocyte adhesion and transmigration
FBLN1	-9.86	Secreted glycoprotein; may be involved in ECM remodeling; downregulated in gastric cancer
FOXA1	-9.82	Forkhead class of DNA binding proteins; loss occurs in aggressive bladder cancer
PPARG	-9.27	Regulate gene transcription together with retinoid X receptors; may influence BCG response
GATA3	-8.97	Transcription factor; downregulated in bladder cancer
CCL15	-8.63	Chemotactic for T cells and monocytes;
TSPAN8	-7.96	Tetraspanin family; interacts with integrins; may regulate motility in various cancers
ADRA2A	-7.75	Alpha-2-adrenergic receptor;
PDGFD	-7.74	PDGF family; can regulate motility in many cancer types and chemotaxis
KRT20	-6.32	Low molecular weight cytokeratin; typically patchy expression in urothelial carcinoma
CYP1A1	-5.7	Hypermethylated in bladder cancer; polymorphisms related to bladder cancer risk
CYP1B1	-5.61	Polymorphisms associated with bladder cancer risk
UGT1A10	-5.28	UDP-glucuronosyltransferase; involved in detoxification of carcinogens
UGT1A7	-5.28	UDP-glucuronosyltransferase; polymorphisms associated with cancer risk

Differentially expressed genes have an adjusted p value of <0.05.

## Discussion

Current pathological classification distinguishes UCa and SCCa as distinct diagnostic entities [4]. This has resulted in numerous publications that have evaluated the differences in clinical outcomes, treatment response and molecular profiles that distinguish these two bladder cancer types, with mixed results [29]. Although some studies have suggested that when compared stage-for-stage, the outcomes are similar for patients with bladder UCa and SCCa [3,29,30], other studies have implicated that SCCa and/or UCa with squamous differentiation may present at a higher stage and behave more aggressively [31-33]. One consistency amongst studies is the limited response of bladder SCCa of the bladder to conventional chemotherapy and/or radiation therapy that is administered in the setting of UCa and may relate to the squamous phenotype [34,35]. To date, however, the relationship between these two forms of bladder cancer that arise from the urothelial lining of the bladder has not been clearly delineated.

The results from our study suggest that UCa generally shares the majority of its dysregulated genes relative to normal urothelium in common with SCCa, with very few uniquely dysregulated genes; in contrast, SCCa – while sharing many genes in common with UCa – shows a much larger category of dysregulated genes that are often in common with SCCa arising at other sites. When considering the relationship between these two closely related entities, two possibilities emerge. First, invasive UCa may represent a default pathway of bladder cancer development, with clonal change resulting in SCCa development and overgrowth of a pre-existent UCa. This hypothesis is supported by the not infrequent finding of mixed morphology bladder cancers, where a well-documented UCa contains areas of squamous and/or glandular differentiation [4]. Further supporting this hypothesis is a prior paper that has examined the relationship of co-existent small cell carcinoma and UCa of the bladder: the results from this prior study suggest that the small cell carcinoma in this setting represented a clonal outgrowth from the background invasive UCa [36] a finding that might not be dissimilar across all other bladder cancer “subtypes” and which can be supported by the findings in this paper. A second possibility is that an early bladder cancer stem cell exists, either prior to invasion or early in the course of invasion, which gives rise to distinct morphological entities along discrete molecular lineages that are considered pure subtypes. Specifically, early molecular changes define a number of shared alterations between various bladder cancer subtypes that subsequently diverge along different morphologic lines [36]. In such a scenario, the limited number of additional alterations identified in UCa would suggest this to be a “default” pathway in bladder carcinogenesis, with significant additional alterations required to develop the squamous phenotype.

Regardless of the model proposed, the current data supports a close evolution between UCa and SCCa, with gene expression changes in the latter primarily reflecting morphological correlates of the squamous phenotype seen in SCCa arising from different sites. Our data also suggest that proliferative changes, including deregulation (and in some cases amplification) of mitotic spindle checkpoint components may be critical in the early stages of bladder tumorigenesis. Further validation of our findings using other “pure” types of bladder cancer – such as adenocarcinoma and small cell carcinoma – will further strengthen the implications of our results, although the rare nature of these other forms of bladder cancer may make such a study challenging.

Although we have used only one technique to analyze the relationship between UCa and SCCa, our ability to reproducibly segregate the entities in our study using both supervised and unsupervised clustering analysis suggest that our data is robust. A second limitation is the use of a limited number of specimens for analysis, although the use of 10 SCCa samples is relatively high given the rarity of this disease entity. The overall gene expression profiles between our two bladder cancer entities suggest that the development of these bladder cancer forms occurs along similar lines. However, it is clear that the magnitude of expression changes differs in some instances; for example, reduction in *ADH1B* occurs by a factor of 40-fold in UCa and 70-fold in SCCa. The importance of relative fold change (versus directionality) in these cancer subtypes was not a primary focus of investigation in this study. However, the relative increase or decrease in mRNA expression may have a relevant biological role when studied in a whole cell system.

Our study has identified numerous categories of genes that may be of relevance to the development of UCa, including mitotic spindle regulators, putative stem cell factors, nicotine metabolizing enzymes and inflammatory regulators. The vast majority of these genes appear to be similarly dysregulated in bladder SCCa. One exception is a large category of inflammatory mediators (*POU2AF1*, *IL33*, *IL6*, *ITK*, *CCL19*) that are altered and may reflect the administration of BCG, which is frequently given intravesically for superficial UCa but not SCCa. In contrast, SCCa appears to overlap significantly in gene expression differences with UCa and additionally contains a large number of additional up- and down-regulated gene products. Perhaps not surprisingly, many of these gene transcripts have been reported in SCCa from the head and neck region, oral cavity, lung and skin. As SCCa is considered to have limited response to therapies conventionally employed for UCa, the broad list of discrete targets – some of which are currently undergoing clinical trials for targeted therapy in other forms of SCCa – may provide an alternative treatment for patients with either pure or mixed SCCa of the bladder.

## Conclusions

In summary, we found that UCa and SCCa of the bladder share a number of differentially regulated genes, suggesting a close evolution of these two major subtypes of bladder cancer. Future studies that seek to further delineate the relationship and, thus, pathogenesis of various forms of bladder cancer will provide additional insight into the development of bladder cancer. Ultimately, the finding of shared molecular changes may allow investigators to develop targeted therapy that may be used either earlier in the course of disease or treat a broader range of cancer morphologies with success.

## Competing interests

The authors declare that they have no competing interests.

## Authors’ contributions

DEH collected the specimens, performed pathologic analysis, interpreted the data and drafted the manuscript; ZZ performed data analysis and drafted the manuscript; DP performed gene expression analysis and reviewed the manuscript; BTT analyzed the data and reviewed the manuscript. All authors read and approve of the final manuscript.

## Pre-publication history

The pre-publication history for this paper can be accessed here:

http://www.biomedcentral.com/1755-8794/6/42/prepub
